# Triclabendazole Sulfoxide Causes Stage-Dependent Embryolethality in Zebrafish and Mouse *In Vitro*

**DOI:** 10.1371/journal.pone.0121308

**Published:** 2015-03-20

**Authors:** Nuria Boix, Elisabet Teixido, Marta Vila-Cejudo, Pedro Ortiz, Elena Ibáñez, Juan M. Llobet, Marta Barenys

**Affiliations:** 1 GRET-CERETOX, INSA-UB and Toxicology Unit, Pharmacology and Therapeutical Chemistry Department, Faculty of Pharmacy, University of Barcelona, Barcelona, Spain; 2 Grup de Mutagènesi, Departament de Genètica i de Microbiologia, Facultat de Biociències, Universitat Autònoma de Barcelona, Bellaterra, Spain; 3 Departament de Biologia Cellular, Fisiologia i Immunologia, Unitat de Biologia Cellular, Facultat de Biociències, Universitat Autònoma de Barcelona, Bellaterra, Spain; 4 Facultad de Ciencias Veterinarias, Universidad Nacional de Cajamarca, Cajamarca, Perú; 5 IUF-Leibniz Research Institute for Environmental Medicine, Düsseldorf, Germany; University of Florida, UNITED STATES

## Abstract

**Background:**

Fascioliasis and paragonimiasis are widespread foodborne trematode diseases, affecting millions of people in more than 75 countries. The treatment of choice for these parasitic diseases is based on triclabendazole, a benzimidazole derivative which has been suggested as a promising drug to treat pregnant women and children. However, at the moment, this drug is not approved for human use in most countries. Its potential adverse effects on embryonic development have been scarcely studied, and it has not been assigned a pregnancy category by the FDA. Thus, to help in the process of risk-benefit decision making upon triclabendazole treatment during pregnancy, a better characterization of its risks during gestation is needed.

**Methodology:**

The zebrafish embryo test, a preimplantation and a postimplantation rodent whole embryo culture were used to investigate the potential embryotoxicity/teratogenicity of triclabendazole and its first metabolite triclabendazole sulfoxide. Albendazole and albendazole sulfoxide were included as positive controls.

**Principal Findings:**

Triclabendazole was between 10 and 250 times less potent than albendazole in inducing dysmorphogenic effects in zebrafish or postimplantation rodent embryos, respectively. However, during the preimplantation period, both compounds, triclabendazole and triclabendazole sulfoxide, induced a dose-dependent embryolethal effect after only 24 h of exposure in rodent embryos and zebrafish (lowest observed adverse effect concentrations = 10 μM).

**Conclusions/Significance:**

In humans, after ingestion of the recommended doses of triclabendazole to treat fascioliasis and paragonimiasis (10 mg/kg), the main compound found in plasma is triclabendazole sulfoxide (maximum concentration 38.6 μM), while triclabendazole concentrations are approximately 30 times lower (1.16 μM). From our results it can be concluded that triclabendazole, at concentrations of the same order of magnitude as the clinically relevant ones, does not entail teratogenic potential *in vitro* during the organogenesis period, but its first metabolite triclabendazole sulfoxide has a high embryotoxic capacity *in vitro* during the preimplantation stage.

## Introduction

*Fascioliasis* and *paragonimiasis* are food-borne trematode diseases affecting millions of people worldwide [1]. They are acquired through ingestion of food contaminated with the larval stages of the parasites and they affect mainly the liver and the lung, respectively. The treatment of choice for these two parasitic diseases is based on triclabendazole (TCBZ) [2, 3, 4], a benzimidazole derivative active against both mature and immature flukes. Although TCBZ is only approved for veterinary use in most countries (except in Egypt, Venezuela and France where it is also registered for human use [5, 6, 7]), it is included in the World Health Organization model list of essential medicines for use in these two parasitic diseases [4], and it has even been suggested as a promising treatment for *Fasciola hepatica* infections occurring in pregnant women and children [8].

TCBZ is regarded as a safe compound during pregnancy, especially in comparison to other benzimidazoles used to treat parasitic diseases. Most of these other benzimidazoles are teratogenic in animals (reviewed in [9]) and have been classified within the Food and Drug Administration (FDA) pregnancy category C (animal reproduction studies have shown an adverse effect on the foetus and there are no adequate and well- controlled studies in humans, but potential benefits may warrant use of the drug in pregnant women despite potential risks). However, this alleged safety relies on a disproportioned difference of available information among the different benzimidazolic compounds. The developmental adverse effects of most benzimidazole derivatives have been comprehensively studied *in vivo* [10, 11, 12, 13, 14, 15] and *in vitro* [16, 17, 18, 19, 20] while the adverse effects of developmental exposure to TCBZ have been scarcely described and extrapolation from the data obtained from the other benzimidazolic compounds is not possible since the mechanism of action of TCBZ seems to be different [21, 22]. There is a single original article available about developmental exposure to TCBZ which describes that it has no teratogenic or embryocidal effects in rats, but the study only covers a third part of the gestation period, leaving the initial and final developmental stages unstudied [23]. The WHO has compiled and published a monography on this drug [24] in which the studies on embryotoxicity and teratogenicity have been summarized. None of the studies of the embryotoxicity and teratogenicity section (2.2.5) covers TCBZ treatment during the whole preimplantation period in any of the species tested (rats, rabbits, sheep and cattle). The reproduction study included (section 2.2.4), which is performed in rats, covers the preimplantation period, but the higher dose administered is much lower than the therapeutical doses given to humans because the study was intended to evaluate TCBZ as a possible residue in food from veterinary administration and not as a product directly administered to humans. Moreover, in the revision of Hurtt [9] TCBZ was classified as fetotoxic in two species, being the rabbits more sensitive than rats, although the original data leading to this classification is not publicly available [9]. Summarizing, there is no public information on *in vitro* studies covering TCBZ treatment during the preimplantation period at human therapeutically relevant doses. Besides, concerning official classifications, TCBZ has not been assigned a pregnancy category by the FDA yet [25]. Thus, to help in the important process of risk-benefit decision making upon TCBZ treatment during pregnancy, a better characterization of TCBZ risks during development, and an *in vivo* reproduction study covering treatment during the preimplantation period at human relevant doses, are needed.

Previous studies with other benzimidazolic compounds like albendazole (ABZ; FDA pregnancy category C) have shown that the parent compound exhibits more teratogenic potential than the first sulfoxide metabolite *in vitro* [16, 17], while the teratogenic effects *in vivo* might be mainly caused by the sulfoxide metabolite due to higher concentrations reached in plasma and the rapid metabolism of the parent compound [16, 26, 27]. Taking this into account, the aims of our study were: 1) to evaluate the developmental toxicity of TCBZ, 2) to study the relationship between the developmental toxic potential of the parent compound and its first metabolite triclabendazole sulfoxide (TCBZSO), 3) to compare TCBZ and ABZ developmental toxic potential, as well as that of their sulfoxide metabolites.

To address the planned objectives the developmental effects of TCBZ and TCBZSO were tested separately using three *in vitro* techniques: the postimplantation whole embryo culture (postWEC), the zebrafish embryo test (ZFET) and the preimplantation whole embryo culture (preWEC). These techniques have been widely used for the assessment of developmental toxicity [28, 29, 30, 31, 32]. They cover early developmental periods before and during the main period of organogenesis, and they allow testing in a whole organism a parent compound and its metabolites independently. ABZ and albendazole sufoxide (ABZSO) were used as positive controls because they are well known teratogenic compounds in animals and they have been studied *in vitro* using the postWEC and the ZFET [16, 19, 20], thus allowing the comparison of previous publications with the present study and with the results of TCBZ and TCBZSO. This strategy has been used before in the study of developmental effects of other benzimidazolic compounds [33], and avoids the bias introduced by the use of different protocols among laboratories when comparing effective concentrations.

Our findings for ABZ and ABZSO correlated with the previous published results, thus confirming the suitability of the approach. TCBZ and TCBZSO developmental toxicity assessment revealed no teratogenic potential for none of them, but a strong embryotoxic potential of TCBZSO at relevant *in vivo* concentrations, being this effect restricted to the preimplantation period.

## Materials and Methods

### Test substances

Triclabendazole, 5-chloro-6-(2,3-dichlorophenoxy)-2-(methylthio)benzimidazole, (TCBZ, = 99.7% purity, CAS number 68786-66-3) and albendazole, methyl-5-(propylthio)-2-benzimidazole-carbamate, (ABZ, ≥ 98% purity, CAS number 54965-21-8) were supplied by Sigma-Aldrich Spain. Triclabendazole sulfoxide (TCBZSO, > 97% purity, CAS number 100648-13-3) and albendazole sulfoxide (ABZSO, > 97% purity, CAS number 54029-12-8) were synthesized by the Organic Chemistry Laboratory from the University of Barcelona (SinteFarma UB). Both sulfoxide metabolites were analysed and proved to be free of parent compound residues.

### Animals

Sprague Dawley rats (Harlan Interfauna Iberica; Barcelona, Spain) and B6CBAF1 (C57BI/6xCBA/J) mice (Charles River, Spain) were kept at a constant dark-light cycle of 12–12 hours (h) and maintained at temperature of 20°C ± 2°C and humidity of 50 ± 10%. Rats and mice were fed with 2014 Teklad Global 14% Protein Rodent Maintenance Diet (Harlan Interfauna Iberica, Barcelona, Spain). Both received tap water *ad libitum*. They were monitored daily for general health.

Adult *Danio rerio* zebrafish (BCN Piscicultura Iberica; Terrassa, Spain) were kept in aquariums with a closed flow-through system in standardized water as specified in ISO 7346-1 and 7346-2 (ISO, 1996; 2 mM CaCl_2_.2H_2_O; 0.5 mM MgSO_4_.7H_2_O; 0.75 mM NaHCO_3_; 0.07 mM KCl). Animals were maintained in an environmentally controlled room: temperature of 26 ± 1°C and constant dark-light cycle of 10–14 h respectively. Females and males were housed separately and fed twice a day with commercial flakes (Tetramin Flakes) and with brine shrimp to stimulate the egg production.

### Ethics Statement

The postWEC and the ZFET studies were approved by the Ethic Committee for Animal Experimentation of the University of Barcelona. The preWEC study was approved by the Ethics Committee on Animal and Human Research of the Autonomous University of Barcelona. All protocols were accepted by the Department of Environment and Housing of the *Generalitat de Catalunya* with the following license numbers, postWEC: DAAM 7148, ZFET: DAAM 7971, preWEC: DAAM 6064, and all were adhered to the *Generalitat de Catalunya* Decree 214/1997 of 30th of July, which regulates the use of animals for experimental and for other scientific purposes.

### Postimplantation whole embryo culture (postWEC)

Nulliparous female rats, which were checked to be in the oestrus phase of the oestrous cycle, were housed with adult males (1:1) for 4 h, considering 2 h of light and 2 h of darkness. Mating was confirmed by the presence of sperm in vaginal smear, and the following 24 h were considered as gestational day 0 (GD 0).

Rat embryos were explanted and evaluated as described before [34] with some variations. Briefly, embryos were explanted at GD 9.5 under sterile conditions. Two embryos were incorporated in flasks filled with 3.5 mL of culture medium which contained 20% of rat serum and 80% of serum mixture (Biochrom AG; Berlin, Germany). TCBZ, TCBZSO, ABZ and ABZSO were dissolved in dimethyl sulfoxide (DMSO; Sigma Aldrich Spain) and added to the culture medium to a final DMSO concentration of 0.1%. Culture flasks were oxygenated at the beginning of the culture with a gas mixture containing 12% O_2_ and after 38 h with a gas mixture containing 50% O_2_. Embryos were cultured at a temperature of 38°C, under rotation at a speed of 25 rpm (Noria R, Ovan Spain) for 48 h. After this period, embryos presenting yolk sac circulation and heartbeat were considered alive and were selected for further evaluation. The yolk sac circulation was evaluated according to the level of formation of blood vessels, giving a score of 1 in the cases in which few vessels were observed or the vascularization was abnormal, a score of 2 when a delay in vascularization was observed and 3 for complete vascularization, as previously described in [35]. Growth evaluation was assessed by crown-rump length and differentiation was evaluated by counting the number of somites and the morphological score. This score is the sum of the scores given to several embryonic structures depending on their differentiation stage, corresponding higher values with higher stages of differentiation [35]. Furthermore, the percentage of dysmorphogenesis was calculated according to [36].

### Zebrafish embryo test (ZFET)

The day before the test, adult male and female zebrafish were transferred (1:1) to breeding tanks (Aquaneering, San Diego, California). Artificial plants and marbles were used as spawning substrate. Spawning and fertilization took place within 30 min after the onset of light in the morning. Eggs were collected and extensively cleaned with ISO-standard 7346 water diluted 1:5 using deionized water. Fertilized eggs were selected under a dissection stereomicroscope (Motic SMZ168, Motic China group, LTD., China). The eggs presenting overt anomalies like asymmetries, formation of vesicles or damaged membranes were discarded.

Exposure and evaluation of eggs was performed as described before [32] with slight variations. At 2 hours post-fertilization (hpf) fertilized eggs were selected and transferred to 6-well plates (Greiner Bio-one, Germany). Ten embryos were randomly distributed into wells and filled with 5 mL of freshly prepared test solutions. TCBZ, TCBZSO, ABZ and ABZSO were dissolved in DMSO and subsequently diluted in 0.3x Danieau’s buffer to a final DMSO concentration of 0.05% (v/v). Embryos were incubated at 26 ± 1°C with a dark-light cycle of 10–14 h for 48 h. Embryolethality was determined at 8 hpf on the basis of egg coagulation, at 26 hpf by the absence of tail detachment or somites formation, and at 50 hpf by the absence of heartbeat. Developmental and teratogenic effects were assessed at 50 hpf by the total morphological score described in [32].

### Preimplantation whole embryo culture (preWEC)

Female mice (6–12 weeks old) were superovulated by intraperitoneal injections of 5 IU of pregnant mare’s serum gonadotropin (Intervet, Spain) and 5 IU of human chorionic gonadotropin (Farma-Lepori, Spain), 48 h apart, and mated with adult males (2:1). Embryos at the one-cell stage were obtained 24 h after the second injection by tearing the oviducts in Hepes-buffered Chatot Ziomek Bavister medium with 150 U/ml hyaluronidase (HCZB) [37] to dissociate the cumulus cells. Denuded embryos were then washed in HCZB and cultured for 96 h in drops of culture medium, potassium simplex optimized medium (KSOM) [38] under oil at 37°C and 5% CO_2_, with or without the compounds of study. TCBZ and TCBZSO dissolved in DMSO were diluted in KSOM medium and the final DMSO concentration in the culture medium was 1 μM. Control embryos were cultured in the presence of 1 μM DMSO. Embryos were observed under the stereomicroscope every 24 h to assess if they were progressing normally until the blastocyst stage or, otherwise, they were arrested in a previous developmental stage. In each observation the number of embryos at the correct developmental stage (two-cells at 24 h, four-cells at 48 h, morula at 72 h and blastocyst at 96 h) were counted.

### Working concentrations

The working concentrations used in postWEC and ZFET techniques were selected from range-finding experiments made for each studied compound. The ABZ and ABZSO range-finding concentrations were chosen from previously published studies about their developmental toxicity [16, 19, 20] and toxicokinetics [13] as well as from studies about toxicokinetics of TCBZ [39]. For the range finding experiments of TCBZ and TCBZSO the selected concentrations were chosen based on the results of ABZ and ABZSO experiments, which were used in this study as positive controls.

For the postWEC experiments, the selected concentrations were 140 μM, 278 μM and 556 μM for TCBZ and 267 μM, 666 μM and 932 μM for TCBZSO. The tested concentrations for ABZ analysis were 0.4 μM, 1.1 μM and 1.9 μM and for ABZSO were 3.4 μM, 9 μM, 12 μM, 14 μM and 16 μM.

The working concentrations for ZFET experiments were 0.1 μM, 0.5 μM, 1 μM, 2.5 μM and 5 μM for TCBZ studies and 0.5 μM, 1 μM, 2.5 μM, 5 μM, 10 μM and 50 μM for TCBZSO. The experiments with ABZ were performed including concentrations of 0.025 μM, 0.05 μM, 0.1 μM, 0.3 μM and 0.5 μM and for ABZSO they included 1 μM, 6 μM, 12 μM, 25 μM and 50 μM.

TCBZ and TCBZSO concentration ranges for the preWEC study were selected based on the results of the ZFET. The tested concentrations for this technique were 1 μM, 3 μM and 10 μM for TCBZ studies and 3 μM, 10 μM, 30 μM and 100 μM for TCBZSO studies.

### Statistics

For the postWEC and ZFET statistical analysis the StatGraphics program was used. The homogeneity of variances of continuous variables was assessed by the Bartlett’s test. Statistical comparisons of homogeneous parameters were made using a one-way analysis of variance, with the ANOVA and the Bonferroni’s test. Statistical comparisons of non-homogeneous parameters were made using the Kruskal-Wallis test. Categorical variables were analysed with the Fisher’s exact test. Quantal data obtained in the preWEC experiments were analysed with the Chi-squared test, using the GraphPad Instat program. In all statistical analyses, a probability of p< 0.05 was considered as statistically significant.

## Results

### Effects of TCBZ and TCBZSO in the rodent postimplantation whole embryo culture

All embryos cultured in the postWEC study presented yolk sac circulation and heartbeat at the end of the culture, except one embryo exposed to ABZSO 14 μM. Therefore, all cultured embryos except this one were selected for further evaluation.

TCBZ exposure for 48 h at 140 μM did not produce any adverse effect in rodent GD 9.5–11.5 embryos (Table 1). This high concentration, far above the *in vivo* relevant ones, was considered the highest concentration at which embryos did not present adverse effects, and was selected as the no observed adverse effects concentration (NOAEC) for TCBZ in the postWEC. At higher concentrations (up to 556 μM), TCBZ induced adverse effects in embryonic growth, assessed by crown-rump length measurement, and in differentiation shown by somites number and total morphological score, being the lowest observed adverse effect concentration (LOAEC) 278 μM. These concentrations also increased the percentage of embryos presenting at least one dysmorphic feature, reaching 100% at 556 μM. In this group of embryos the number of somites could not be determined due to widespread abnormalities (abbreviated in the table as n.c.: not possible to count). The most observed abnormalities were in the yolk sac, branchial bars, flexion, head and optic vesicles (S1 Table). Detailed descriptions of all abnormalities assessed in the postWEC are described in S2 Table.

**Table 1:** Effects of TCBZ and TCBZSO in the rodent postimplantation whole embryo culture.

		E	Yolk sac circulation	Crown-rump length	Somites number	Morphological score	Dysmorphogenic embryos
			Median	mm ± SD	Mean ± SD	Mean ± SD	%
**Control**		76	3.0	3.7 ± 0.6	26.8 ± 1.9	39.1 ± 2.6	2.6
	140 μM	8	3.0	3.5 ± 0.2	26.9 ± 1.1	39.0 ± 1.5	0
**TCBZ**	278 μM	8	1.0*	3.2 ± 0.5	25.7 ± 2.0*	35.1 ± 5.9*	62.5*
	556 μM	8	1.0*	2.6 ± 0.6*	n.c.	21.0 ± 3.0*	100*
	267 μM	9	3.0	3.4 ± 0.4	26.6 ± 1.6	37.9 ± 2.8	0
**TCBZSO**	666 μM	9	3.0*	3.1 ± 0.7*	25.1 ± 2.1*	30.8 ± 7.1*	55.6*
	932 μM	8	1.0*	2.3 ± 0.5*	n.c.	20.3 ± 2.7*	100*
	0.4 μM	8	3.0	3.5 ± 0.1	25.8 ± 1.9	38.9 ± 0.6	0
**ABZ**	1.1 μM	8	3.0	3.2 ± 0.2	23.9 ± 1.1*	30.8 ± 2.1*	50*
	1.9 μM	8	1.0*	2.9 ± 0.4*	21.9 ± 4.5*	26.4 ± 5.2*	100*
	3.4 μM	8	3.0	3.6 ± 0.2	26.9 ± 1.6	38.5 ± 2.1	12.5
	9 μM	13	3.0	3.4 ± 0.3	25.0 ± 2.8	35.8 ± 5.2	30.8*
**ABZSO**	12 μM	10	3.0	3.6 ± 0.2	25.5 ± 2.0	38.6 ± 1.2	40*
	14 μM	11	2.0*	3.4 ± 0.2	22.9 ± 1.6*	29.7 ± 6.6*	72.7*
	16 μM	11	1.0*	3.1 ± 0.6*	19.3 ± 2.4*	21.8 ± 6.7*	100*

Total number of embryos (E), yolk sac circulation, crown-rump length, number of somites, total morphological score and percentage of dysmorphogenesis obtained in each concentration group. SD: standard deviation, n.c.: not possible to count

*: p< 0.05.

TCBZSO did not induce any adverse effects in embryonic growth or development at 267μM. Thus, this concentration accounting for approximately seven times the *in vivo* relevant concentrations in humans, was selected as the NOAEC for TCBZSO in the postWEC. The embryos incubated at higher concentrations of TCBZSO (LOAEC = 666 μM) presented a dose-dependent significant decrease in the total morphological score and crown-rump length (Table 1). Besides, a dose-dependent significant increase in the percentage of abnormalities was observed. The main dysmorphogenic effects induced by TCBZSO were in the yolk sac, branchial bars, flexion, head and optic and otic vesicles (S1 Table).

To confirm the validity of these results, and thus the absence of teratogenic potential of TCBZ and TCBZSO *in vitro*, a pair of positive control compounds belonging to the same chemical class, ABZ and ABZSO, was used for comparison. ABZ and ABZSO decreased the somites number and the total morphological score and increased the percentage of dysmorphogenesis dose-dependently (Table 1). The LOAECs were obtained at *in vivo* relevant concentrations and were as low as 1.1 and 9 μM respectively. All embryos incubated at the highest ABZ concentration (1.9 μM) presented dysmorphogenesis, mainly in yolk sac, in branchial bars, head, heart and caudal part and presence of subcutaneous blisters (S1 Table). ABZSO exposure also produced subcutaneous blisters and a dose-dependent correlation for branchial bars, head and caudal part dysmorphogenesis. Other main abnormalities presented after ABZSO treatment were in the yolk sac, flexion, heart and optic and otic vesicles (S1 Table).

Summarizing the postWEC results, TCBZ and TCBZSO exposure between GD 9.5 and 11.5 did not induce adverse effects in rodent embryos until concentrations at least approximately seven times higher than the *in vivo* relevant ones, indicating that these compounds have a rather low teratogenic potential at *in vivo* relevant concentrations.

### Effects of TCBZ and TCBZSO in the zebrafish embryo test

Zebrafish embryos incubated with TCBZ at 1 μM did not present any adverse effects. Higher TCBZ exposure produced an effect on the zebrafish embryonic differentiation (LOAEC = 2.5 μM), assessed by a significant decrease on the total morphological score (Table 2). It also produced a significant increase in the percentage of embryos presenting at least one dysmorphic feature (LOAEC = 2.5 μM). All embryos incubated with TCBZ at the concentration of 5 μM were dead at 50 hpf. The most observed abnormalities in zebrafish embryos exposed to TCBZ are detailed in S3 Table.

**Table 2:** Effects of TCBZ and TCBZSO in the zebrafish embryo test.

		N	E	Lethality	Morphological score	Dysmorphogenic embryos
				%	Mean ± SD	%
**Control**		14	140	5	35.9 ± 0.2	1.6
	0.1 μM	3	30	0	34.8 ± 1.8	6.7
	0.5 μM	3	30	0	34.8 ± 0.0	13.3
**TCBZ**	1 μM	3	30	0	35.8 ± 0.4	13.3
	2.5 μM	3	30	0	33.0 ± 3.4*	60*
	5 μM	3	30	100*	n.d.	n.d.
	0.5 μM	3	30	17	36.0 ± 0.0	0
	1 μM	3	30	10	36.0 ± 0.0	0
**TCBZSO**	2.5 μM	3	30	20	36.0 ± 0.0	0
	5 μM	3	30	7	36.0 ± 0.0	0
	10 μM	4	40	35*	36.0 ± 0.0	0
	50 μM	3	30	100*	n.d.	n.d.
	0.025 μM	3	30	7	36.0 ± 0.0	0
	0.05 μM	3	30	0	35.9 ± 0.2	3.3
**ABZ**	0.1 μM	4	40	0	35.9 ± 0.2	2.5
	0.3 μM	3	30	17*	25.5 ± 2.1*	72*
	0.5 μM	3	30	100*	n.d.	n.d.
	1 μM	3	30	0	36.0 ± 0.0	0
	6 μM	3	30	0	35.7 ± 0.9	3.3
**ABZSO**	12 μM	3	30	0	35.5 ± 0.6	10
	25 μM	3	31	13	18.0 ± 0.0*	100*
	50 μM	3	30	100*	n.d.	n.d.

Number of independent experiments (N), total number of embryos (E), percentage of lethality, total morphological score and percentage of dysmorphogenesis obtained in each concentration group. SD: standard deviation, n.d.: not determined

*: p< 0.05.

TCBZSO exposure also induced an embryolethal effect at 26 hpf, but starting at 10 μM concentrations (LOAEC = 10 μM). None of the studied concentrations of TCBZSO decreased the total morphological score or induced any dysmorphogenesis on the treated embryos (Table 2). Thus, TCBZSO NOAEC was established at 5 μM.

To compare the embryotoxic potential of TCBZ and TCBZSO with previously studied compounds of the same family, ABZ and ABZSO were used as positive control compounds. ABZ and ABZSO produced a significant increase in the percentage of lethality in the higher concentrations, reaching 100% at 0.5 and 50 μM respectively. ABZ and ABZSO produced a significant effect in the embryonic differentiation expressed as a decrease in the total morphological score at the highest concentrations in which embryos were still alive, 0.3 and 25 μM respectively. At this concentrations a significant increase in the percentage of embryos presenting dysmorphogenesis, was also observed for both compounds, being of 100% in the case of ABZSO (S3 Table).

To sum up, the ZFET results showed that TCBZ exposure until 50 hpf produced a significant effect in embryonic differentiation and in lethality at concentrations 2.5 and 5 μM, respectively. On the other hand, TCBZSO exposure did not produce any effects in embryonic growth or differentiation, but produced a significant embryolethal effect during the first 24 h of exposure at relevant *in vivo* concentrations, starting at 10 μM, and therefore rising a concern about the embryotoxic potential of TCBZSO during the first stages of development.

### Effects of TCBZ and TCBZSO in the rodent preimplantation whole embryo culture

To confirm if embryolethal effects of TCBZSO observed in zebrafish embryos were also relevant for mammalian embryos, a 96 h mouse embryo preimplantation culture, checking embryonic development every 24 h, was performed. In this case, the analysis of TCBZ and TCBZSO was performed via blind studies, and ABZ and ABZSO were not used as positive controls due to the absence of previous studies analysing the effects of these compounds during the rodent preimplantation period *in vitro*.

TCBZ exposure did not produce any significant effect in preimplantation embryos at 3 μM (Table 3). Nonetheless, it produced a significant increase in the percentage of lethality of the highest studied concentration group (LOAEC = 10 μM), which started after only 24 h of exposure.

**Table 3:** Effects of TCBZ and TCBZSO in the rodent preimplantation whole embryo culture.

		N	E	2-cells 24 h	4-cells 48 h	Morula 72 h	Blastocyst 96 h
				%	%	%	%
**Control**		4	48	2.1	4.2	6.3	22.9
	1 μM	4	48	4.2	8.3	8.3	18.8
**TCBZ**	3 μM	4	48	6.3	6.3	10.4	20.8
	10 μM	3	49	18.4*	24.5*	24.5*	36.7
	3 μM	3	48	4.2	14.6	16.7	31.3
**TCBZSO**	10 μM	3	49	24.5*	30.6*	36.7*	55.1*
	30 μM	3	49	24.5*	79.6*	95.9*	98*
	100 μM	1	15	100*	100*	100*	100*

Number of independent experiments (N), total number of embryos (E), percentage of lethality in every developmental stage for each concentration group.

*: p< 0.05

TCBZSO did not induce adverse effects at 3 μM. Starting at 10 μM (LOAEC), TCBZSO produced a significant concentration-dependent lethal effect, which was already evident during the first 24 h of exposure. At 30 μM, which is a concentration still relevant after parasitosis treatment in humans, TCBZSO produced lethality at higher rates than 50% during the first 48 h of culture.

**Fig 1:**
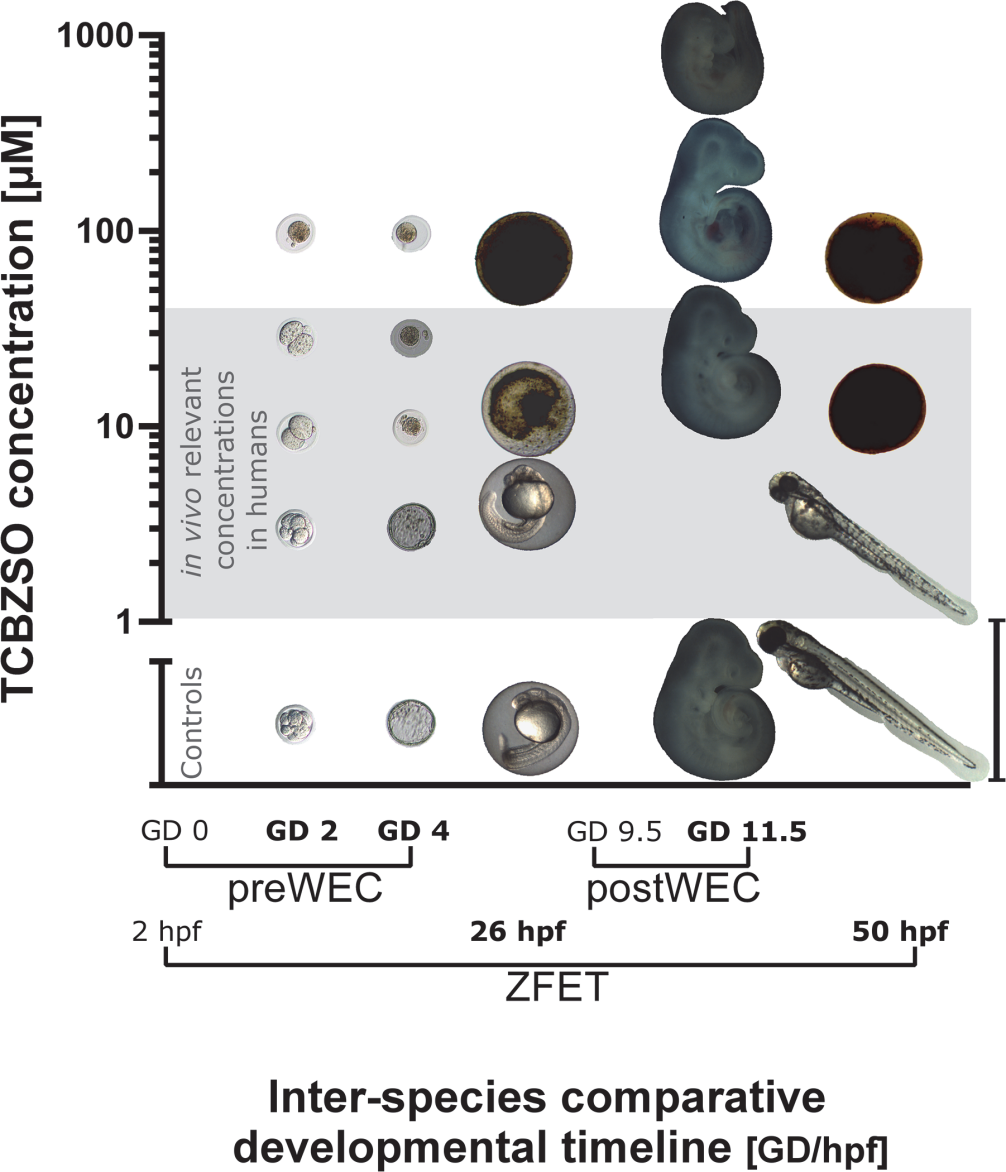
Graphical summary comparing TCBZSO results across species and developing time. Representative pictures of rodent embryos exposed to increasing concentrations of TCBZSO from GD 0 to GD 4 in the preWEC culture (LOAEClethality = 10 μM) and from GD 9.5 to GD 11.5 in the postWEC culture (LOAECdysmorphogenesis = 666 μM). Zebrafish embryos were exposed to TCBZSO from 2 hpf to 50 hpf, a developmental period comprising the stages covered by both rodent cultures. No dysmorphogenesis were observed (maximum concentration tested = 50 μM), but TCBZSO was embryolethal during the first 24 h of culture (LOAEClethality = 10 μM). Pictures correspond to the developmental time points marked in bold in the x-axis. PreWEC embryos pictures are 10 times magnified respect to postWEC and zebrafish embryos pictures. Scale bar: 4 mm for postWEC and zebrafish embryos; and 400 μm for preWEC embryos. ZFET: zebrafish embryo test (concentrations = 0, 5, 10 and 50 μM); preWEC: preimplantation whole embryo culture (concentrations = 0, 3, 10, 30 and 100 μM); postWEC: postimplantation whole embryo culture (concentrations = 0, 27, 267 and 666 μM); GD: gestational day; hpf: hours post-fertilization.

Consequently, the preWEC results confirmed a strong embryotoxic potential of TCBZSO also in mammalian embryos *in vitro* (For a graphical overview on the results of TCBZSO in the three *in vitro* techniques used, see Fig. 1).

## Discussion

*Fascioliasis* and *paragonimiasis* are parasitic infections caused by flatworms or flukes. They are classified within the foodborne trematodiases group, which is included among the major neglected tropical diseases. Millions of people affected by these parasitoses are in need of effective and safe therapies, and among them women at childbearing age represent an important portion of population. Direct exclusion of all these women from massive treatment programs to avoid exposure during pregnancy would prevent their access to treatment for a large proportion of their reproductive lives. Thus, it is of uppermost importance to correctly characterize the risks associated with TCBZ treatment during pregnancy, and compile the maximum information on concentrations of the same order of magnitude as the clinically relevant ones, to identify if there are developmental periods where the risk associated with the exposure is lower than in others.

*In vivo*, TCBZ concentrations found in plasma after its administration are very low due to its rapid metabolism [22]. For this reason it is also important to study the effects of its main metabolite TCBZSO, which achieves higher concentrations in plasma. For example in rabbits, the TCBZSO maximum plasma concentration (C_max_) after a single 10 mg/kg TCBZ dose is 33 μM (transformed to μM from [40]). Apart from the data coming from laboratory animals, several studies have evaluated the relevant concentrations after TCBZ therapeutical administration to food producing animals and humans. In sheep, TCBZ doses of 10 mg/kg resulted in 37.26 μM C_max_ of TCBZSO (calculated from [41]), and in cows after a 12 mg/kg dose, the C_max_ was 71.34 μM (calculated from [39]). In humans, after a therapeutical dose of 10 mg/kg TCBZ, the TCBZ C_max_ was 1.16 μM ([42] and reviewed by [22]) and the TCBZSO C_max_ was 26.6 μM (calculated from [43]) or between 15.8 μM and 38.6 μM depending on the concomitant food intake situation ([44] and reviewed by [22]). *In vivo* relevant concentrations of ABZ and ABZSO in food producing animals and in humans have recently been reviewed by Eckardt [16].

In our studies with rodent embryos exposed during the postimplantation period, the teratogenic potential of TCBZ was approximately 250 times lower than the potential of ABZ (Table 4). Both sulfoxide metabolites had less teratogenic potential than their respective parent compounds, correlating with previous observations with other benzimidazolic derivatives [16, 17]. In this case, TCBZSO was 2.5 times less potent than its parent compound (Table 4). Even if it occurred at very different concentration ranges, the four tested compounds induced abnormal head and abnormal branchial bars in rodent embryos exposed during organogenesis. These adverse effects are characteristic of developmental exposure to benzimidazoles, being the second effect also representative of other azolic derivatives like triazoles [45]. Besides these alterations, ABZ and ABZSO exposed embryos exhibited very evident subcutaneous blisters in the facial laterals, abnormal caudal part and abnormal heart (S1 Table). Although TCBZ and TCBZSO have the ability of producing the same dysmorphogenesis as other compounds of their family, these effects occur at concentrations much higher than those achieved *in vivo* in humans after parasitosis treatment, indicating that neither TCBZ nor TCBZSO have teratogenic potential at the actual recommended therapeutical doses.

**Table 4:** Summary of LOAEC values of rodent and zebrafish embryos exposed to TCBZ, TCBZSO, ABZ and ABZSO during 48 h in comparison to the available *in vitro* and *in vivo* literature.

					*In vitro literature*			*In vivo literature*
	postWEC	ZFET	ZFET	preWEC	postWEC	ZFET	preWEC	
	LOAEC (μM)	LOAEC dysmorphogenesis (μM)	LOAEC lethality (μM)	LOAEC (μM)	LOAEC (μM)	LOAEC (μM)	LOAEC (μM)	Plasma concentrations at teratogenic doses (μM)
**TCBZ**	278	2.5	5	10	n.a.	n.a.	n.a	not teratogenic at a 200mg/kg dose [23]
**TCBZSO**	666	not dysmorphogenic	10	10	n.a.	n.a.	n.a.	n.a.
**ABZ**	1.1	0.3	0.3	n.a.	3.7 [16]	0.3 [19, 20]	n.a.	0.94 ([47]; reviewed and transformed to μM by [13])
**ABZSO**	9	25	50	n.a.	17.7 [16]	6.8 [20] Non observed adverse effects [19]	n.a.	12.8 ([47]; reviewed and transformed to μM by [16])

LOAEC: lowest observed adverse effect concentration, n.a.: data not available.

The results of the zebrafish embryo test reproduced those of the postWEC concerning dysmorphogenic potential. Again, ABZ was more potent than ABZSO at concentrations in good agreement with the published literature (0.3 μM), and TCBZ was more potent than TCBZSO which was not dysmorphogenic at all (Table 4). However, TCBZSO concentrations causing embryolethality were five times lower than ABZSO concentrations killing the embryos or 2.5 times lower than concentrations producing malformations (Table 4). These results show no dysmorphogenic activity but point to a remarkable embryotoxic potential of TCBZSO. In our test, compound exposure started at 2 hpf, leaving the very first stages of development untreated. As previous studies observed higher sensitivity to ABZ during the first stages of development, it cannot be excluded that TCBZSO has higher embryotoxicity when exposure starts at 0 hpf. After 48 h of exposure, the observed effects for all compounds in the zebrafish embryos were of general toxicity, as for example cardiac oedema or decreased pigmentation and there were no evident characteristic dysmorphogenesis for each one. Other studies working with zebrafish and azolic derivatives showed that the ZFET could correctly classify the potency of the compounds but could not reproduce the *in vivo* observed specific dysmorphogenesis [45].

The differences in embryolethality observed in the ZFET and the postWEC TCBZSO results could be due to 1) inter-species differences in susceptibility to the compound or in compound availability, or to 2) different susceptibility of the early and middle developmental periods after exposure to TCBZSO, as the ZFET covers earlier developmental stages than the postWEC (Fig. 1).

To distinguish between these two options, another rodent culture experiment was performed with TCBZ and TCBZSO but in this case covering only the earliest period of development (from GD 0 to GD 4). And indeed, after only 24 h of exposure TCBZSO caused a significant increase in lethality at concentrations as low as 10 μM, thus confirming the relevance of the high embryotoxic potential of TCBZSO in mammals. Previously published mechanistic effects of this compound like protein synthesis inhibition or microtubule inhibition in fluke vitelline cells [46] could be related to TCBZSO effects in preimplantation embryos, but further studies need to be done to elucidate the exact mechanisms by which TCBZSO exerts its embryotoxicity in mammals. Besides, studies exposing pregnant rodent dams during the preimplantation period are required to confirm the stage-dependent embryotoxic potential of TCBZSO *in vivo*. On the other hand, TCBZ only increased lethality in preimplantation embryos, at concentrations 10 times higher than the *in vivo* relevant ones.

From our results it can be concluded that TCBZ, at concentrations of the same order of magnitude as those achieved after intake of the recommended treatment doses for *fascioliasis* and *paragonimiasis*, does not entail relevant dysmorphogenic potential *in vitro* during the organogenesis period, but its first metabolite TCBZSO has a high embryotoxic capacity *in vitro* during the preimplantation stage.

## Supporting Information

S1 Table Frequency (%) of dysmorphogenesis observed in postWEC experiments. (DOCX)

S2 Table Description of the abnormalities observed in the embryos cultured using the postWEC technique. (DOCX)

S3 Table Frequency (%) of dysmorphogenesis observed in ZFET experiments. (DOCX)
