# Supplementary material for: Triclabendazole Sulfoxide Causes Stage-Dependent Embryolethality in Zebrafish and Mouse In Vitro
Source: PLoS One. 2015 Mar 20;10(3):e0121308. doi: 10.1371/journal.pone.0121308 (PMC4368200; doi:10.1371/journal.pone.0121308)
Supplement: S1 Table — (DOCX) [file pone.0121308.s001.docx]

S1 Table: Frequency (%) of dysmorphogenesis observed in postWEC experiments.

|  |  | Yolk sac | Subcutaneous blisters | Branchial bars | Flexion | Head | Heart | Caudal part | Optic vesicles | Otic vesicles | Limbs |
| --- | --- | --- | --- | --- | --- | --- | --- | --- | --- | --- | --- |
|  | Control | 2.6 | ---- | ---- | 1.3 | ---- | ---- | ---- | ---- | ---- | ---- |
|  | 140 µM | ---- | ---- | ---- | ---- | ---- | ---- | ---- | ---- | ---- | ---- |
| **TCBZ** | 278 µM | 62.5 | ---- | 25 | 12.5 | 12.5 | ---- | ---- | ---- | 12.5 | ---- |
|  | 556 µM | 100 | ---- | 100 | 50 | 75 | 12.5 | ---- | 37.5 | ---- | ---- |
|  | 267 µM | ---- | ---- | ---- | ---- | ---- | ---- | ---- | ---- | ---- | ---- |
| **TCBZSO** | 666 µM | 33.3 | ---- | 11.1 | ---- | 33.3 | ---- | ---- | 33.3 | ---- | ---- |
|  | 932 µM | 100 | ---- | 100 | 87.5 | 75 | ---- | ---- | 62.5 | 25 | 12.5 |
|  | 0.4 µM | ---- | ---- | ---- | ---- | ---- | ---- | ---- | ---- | ---- | ---- |
| **ABZ** | 1.1 µM | 25 | 50 | 50 | ---- | ---- | 12.5 | ---- | ---- | ---- | ---- |
|  | 1.9 µM | 75 | 37.5 | 100 | 12.5 | 62.5 | 37.5 | 37.5 | ---- | ---- | ---- |
|  | 3.4 µM | ---- | ---- | ---- | ---- | ---- | ---- | ---- | ---- | ---- | ---- |
|  | 9 µM | 7.7 | 30.8 | 23.1 | 7.7 | ---- | 7.7 | 7.7 | ---- | ---- | ---- |
| **ABZSO** | 12 µM | ---- | 40 | 10 | ---- | 40 | ---- | ---- | ---- | ---- | ---- |
|  | 14 µM | 18.2 | 54.5 | 63.6 | 9.1 | 36.4 | ---- | 36.4 | 9.1 | 36.4 | ---- |
|  | 16 µM | 63.6 | 45.5 | 100 | 63.6 | 72.7 | 45.5 | 72.7 | 27.3 | 45.5 | 18.2 |
