# Supplementary material for: Triclabendazole Sulfoxide Causes Stage-Dependent Embryolethality in Zebrafish and Mouse In Vitro
Source: PLoS One. 2015 Mar 20;10(3):e0121308. doi: 10.1371/journal.pone.0121308 (PMC4368200; doi:10.1371/journal.pone.0121308)
Supplement: S2 Table — (DOCX) [file pone.0121308.s002.docx]

S2 Table: Description of the abnormalities observed in the embryos cultured using the postWEC technique.

| **Type of dysmorphogenesis** | **Definition of the dysmorphogenesis** |
| --- | --- |
| Yolk sac | The yolk sac did not present a rounded shape or showed a vessels defect |
| Subcutaneous blisters | The embryo presented a blister at one or both facial sides |
| Branchial bars | The branchial bars appeared fused or showed a decrease in size |
| Flexion | The curvature of the embryo was directed to the posterior side |
| Head | Abnormal proportion of forebrain, mid- and hind-brain sizes or deformed due to the opening of the cranial neural pore and the protrusion of tissue |
| Heart | Abnormal shape of the heart, accumulation of fluid in the cardiac region with abnormal increased size of the heart cavity |
| Caudal part | The caudal part of the embryo was curved, deformed or absent |
| Optic vesicles | Abnormal shape of at least one optic vesicle or abnormal narrowing at the central part |
| Otic vesicles | Abnormal shape of at least one otic vesicle or abnormal narrowing at the central part |
| Limbs | The limbs were not present |
