# Supplementary material for: Triclabendazole Sulfoxide Causes Stage-Dependent Embryolethality in Zebrafish and Mouse In Vitro
Source: PLoS One. 2015 Mar 20;10(3):e0121308. doi: 10.1371/journal.pone.0121308 (PMC4368200; doi:10.1371/journal.pone.0121308)
Supplement: S3 Table — (DOCX) [file pone.0121308.s003.docx]

S3 Table: Frequency (%) of dysmorphogenesis observed in ZFET experiments.

|  |  | Developmental delay | Cardiac oedema | Brain necrosis | Eye pigmentation | Head-body pigmentation | Movement | Flexion |
| --- | --- | --- | --- | --- | --- | --- | --- | --- |
|  | Control | ---- | ---- | ---- | ---- | ---- | ---- | 1.6 |
|  | 0.1 µM | 6.7 | ---- | ---- | ---- | 6.7 | ---- | 3.3 |
| **TCBZ** | 0.5 µM | ---- | 13.3 | ---- | ---- | ---- | ---- | ---- |
|  | 1 µM | ---- | 10 | 10 | ---- | ---- | ---- | 6.7 |
|  | 2.5 µM | 50 | ---- | ---- | ---- | 50 | ---- | 20 |
|  | 0.5 µM | ---- | ---- | ---- | ---- | ---- | ---- | ---- |
|  | 1 µM | ---- | ---- | ---- | ---- | ---- | ---- | ---- |
| **TCBZSO** | 2.5 µM | ---- | ---- | ---- | ---- | ---- | ---- | ---- |
|  | 5 µM | ---- | ---- | ---- | ---- | ---- | ---- | ---- |
|  | 10 µM | ---- | ---- | ---- | ---- | ---- | ---- | ---- |
|  | 0.025 µM | ---- | ---- | ---- | ---- | ---- | ---- | ---- |
| **ABZ** | 0.05 µM | ---- | 3.3 | ---- | ---- | ---- | ---- | ---- |
|  | 0.1 µM | ---- | 2.5 | ---- | ---- | ---- | ---- | ---- |
|  | 0.3 µM | 72 | 72 | ---- | 16 | 40 | 32 | ---- |
|  | 1µM | ---- | ---- | ---- | ---- | ---- | ---- | ---- |
| **ABZSO** | 6µM | ---- | 3.3 | ---- | ---- | ---- | ---- | ---- |
|  | 12µM | ---- | 10 | ---- | ---- | ---- | ---- | ---- |
|  | 25µM | 100 | 100 | 100 | 100 | 100 | 100 | ---- |
